# Spontaneous intraoperative lumbar fracture leading to an unexpected correction in ankylosing spondylitis corrective surgery – a case report

**DOI:** 10.3205/iprs000148

**Published:** 2020-11-24

**Authors:** Christoph-Eckhard Heyde, Stefan Glasmacher, Nicolas H. von der Höh, Anna Völker

**Affiliations:** 1 Spine Division, Department of Orthopedics, Trauma and Plastic Surgery, University of Leipzig, Germany

**Keywords:** ankylosing spondylitis, spine, corrective surgery, spontaneous fracture

## Abstract

Severe kyphotic deformity in patients with ankylosing spondylitis can be corrected surgically to achieve a better spinal alignment and an improved visual axis. Different surgical techniques are used today depending on the extent of ossification and the degree of kyphosis. It is well known that the underlying disease leads to distinct biomechanical changes of the spinal column causing an increased fracture risk especially in case of minor trauma. This includes manipulations during surgical procedures as well as during the required perioperative measures.

We present the case of a 45-year-old patient with severe global kyphotic deformity due to ankylosing spondylitis. During the elective corrective surgery (pedicle subtraction osteotomy at the level of L3) the patient sustained a spontaneous fracture at L2/3. This fortunately nondisplaced wedge-shaped fracture in the sense of a Smith-Peterson osteotomy led to a spontaneous correction of the kyphosis. The described unexpected event required a change in the surgical strategy. Correction could be achieved using a two-stage surgical procedure without further drawbacks for the patient.

This case report stresses the need of particular attention regarding the increased susceptibility of the spinal column in case of ankylosing spondylitis.

## Zusammenfassung

Korrekturosteotomien an der Wirbelsäule bei M. Bechterew führen bei ausgeprägten kyphotischen Deformitäten zu einer Verbesserung des sagittalen Profils und der Blickachse. Dabei kommen in Abhängigkeit vom Schweregrad der Kyphose und dem Ausmaß der Ossifikation verschiedene operative Techniken zum Einsatz. Die zu Grunde liegende Erkrankung führt zu einer ausgeprägten Veränderung der biomechanischen Eigenschaften der Wirbelsäule und damit zu einem deutlich erhöhten Frakturrisiko. Schon Bagatelltraumata können Frakturen hervorrufen, gefährdet ist die Wirbelsäule bei M. Bechterew auch durch intraoperative und perioperative Maßnahmen.

Vorgestellt wird der Fall eines 45-jährigen Patienten mit einer ausgeprägten Globalkyphose bei M. Bechterew. Während der erforderlichen elektiven Korrekturoperation (Pedikelsubtraktions-Osteotomie Höhe L3) kam es zu einer spontanen Fraktur in Höhe der verknöcherten Bandscheibe L2/3. Dadurch kam es zu einem spontanen keilförmigen Aufgehen der ventralen Wirbelanteile ohne weitere Dislokation und damit zu einer spontanen Korrektur im Sinne einer Smith-Peterson-Osteotomie. Daraus resultierte ein intraoperativer Wechsel der chirurgischen Vorgehensweise. Die Korrektur konnte dann durch ein zweizeitiges Vorgehen erreicht werden.

Dieser Fall unterstreicht die notwendige besondere Aufmerksamkeit bezüglich der erhöhten Frakturanfälligkeit der Wirbelsäule bei Patienten mit M. Bechterew.

## Background

Ankylosing spondylitis (Bechterew’s disease) is a chronic progressive rheumatic spondyloarthritis. One of the main manifestation sites of this disease are the spinal column and the sacroiliac joint, additional peripheral manifestations are common. The etiology remains unknown, genetic predisposing factors are described. Usually the disease manifests between the 20^th^ and 40^th^ year of age. Men are more frequently affected than women. Pain, a gradual loss of mobility and both ankylosing of the spine and development of global sagittal imbalance due to progressive kyphosis are typical for this disease [1], [2].

The underlying rheumatic disease is responsible for many changes which affect the spinal column. Different processes like ossification, development of osteoporosis and subsequent changes in the spinal alignment lead to changes of the biological and biomechanical properties of the spinal column. Chronic pain, loss of mobility, the disturbed sagittal spinal alignment, and the resulting loss of the normal visual axis are restrictions for the affected patients. In addition, these patients have an increased tendency to fall, based on the before mentioned changes [1], [2], [3], [4], [5], [6], [7]. 

Corrective osteotomies at the spinal column are an option to improve the sagittal balance and the visual axis. Different surgical techniques are described depending on the localization of the main kyphosis, the degree of spinal ossification and the sagittal spinal imbalance [8]. These procedures can be challenging. However, the results regarding the improvement of both the sagittal balance and the visual axis are particularly good [8], [9]. Complications should be considered. The frequency of complications increases with the age of the patients and with the dimension of the required surgical procedure [10].

The biomechanical changes due to the underlying disease lead to an increased susceptibility of the spinal column to fractures not only in case of high-velocity trauma, but also in case of minor trauma. The progressive ossification of the whole spine results in a completely stiff spine with biomechanical properties comparable to a long bone with long lever arms. This condition, the subsequent osteoporosis, the kyphotic deformity, as well as the changes in the muscles of the spine (sarcopenia) result in the mentioned significant increased fracture susceptibility to minor trauma [3], [4], [5], [6], [7]. Consequently, spontaneous fractures during surgical procedures and during the necessary perioperative measures are described in the literature [11]. Therefore, brusque maneuvers during surgical procedures and the required perioperative measures like the patient’s repositioning should be avoided. This group of patients requires special attention during the entire treatment phase based on a comprehensive knowledge about the underlying disease.

In the following, we present the case of a spontaneous intraoperative fracture during corrective surgery in case of severe kyphosis in a patient with ankylosing spondylitis. 

## Case description

A 45-year-old man with ankylosing spondylitis, resulting in global kyphosis with a sagittal spinal imbalance and a restricted visual axis was planned for a corrective osteotomy. The ossification of the spine was complete including the sacroiliac joints. An old consolidated fracture at the level L1 led to an increase of the global malalignment. Ankylosing spondylitis was known and treated since the age of 26. In the last five years, the patient was treated with non-steroidal anti-inflammatory drugs and exercises. His complaints were posture-dependent pain of the hip joints and lack of the possibility to compensate the disturbed visual axis. 

**Figure 1:**
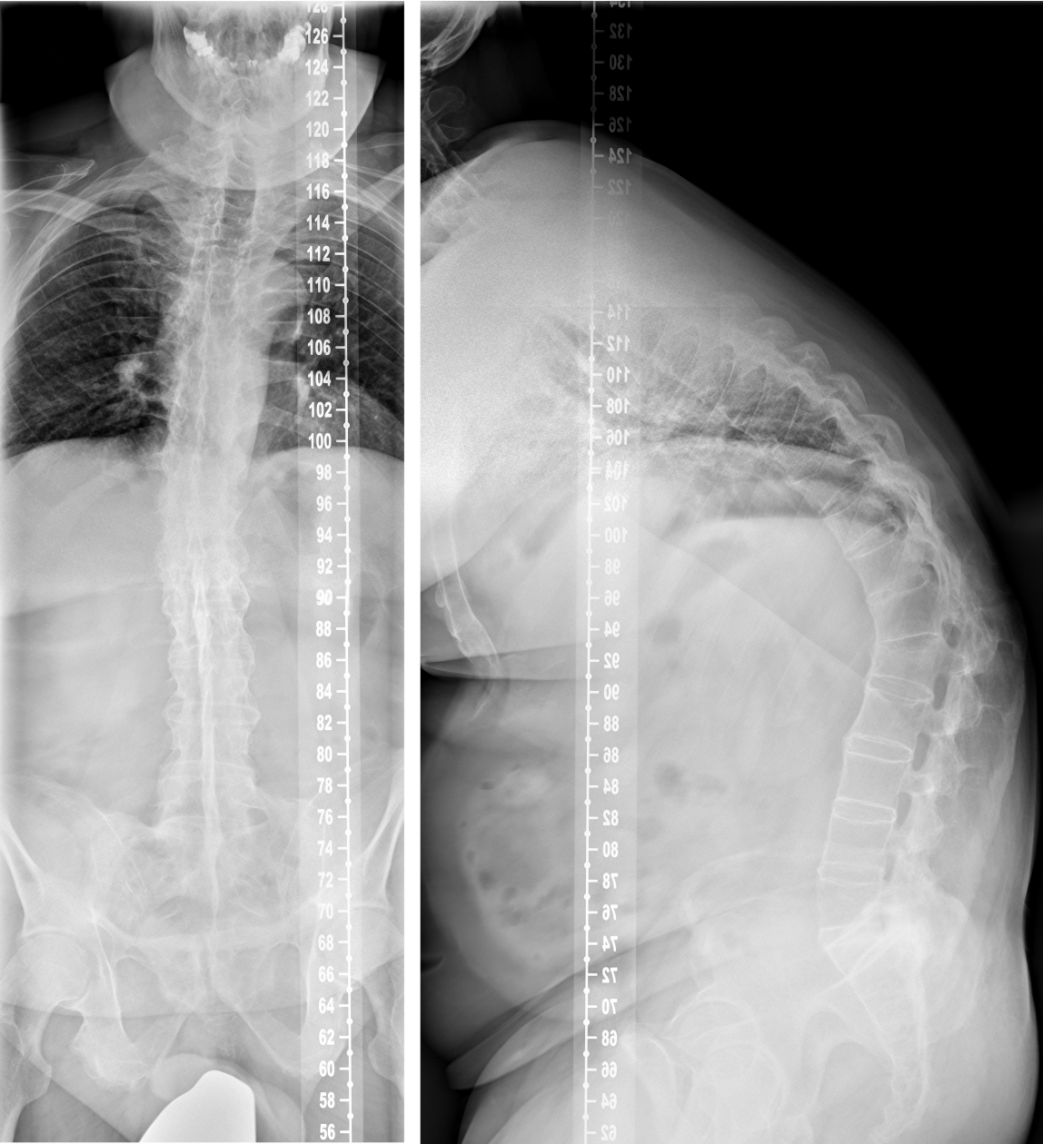
Conventional X-rays of the whole spine in two planes in standing position (ap-view left and lateral view right) revealing the ossification and the kyphotic deformity of the whole spine with sagittal imbalance as well as the reduced bone mineral density.

**Figure 2:**
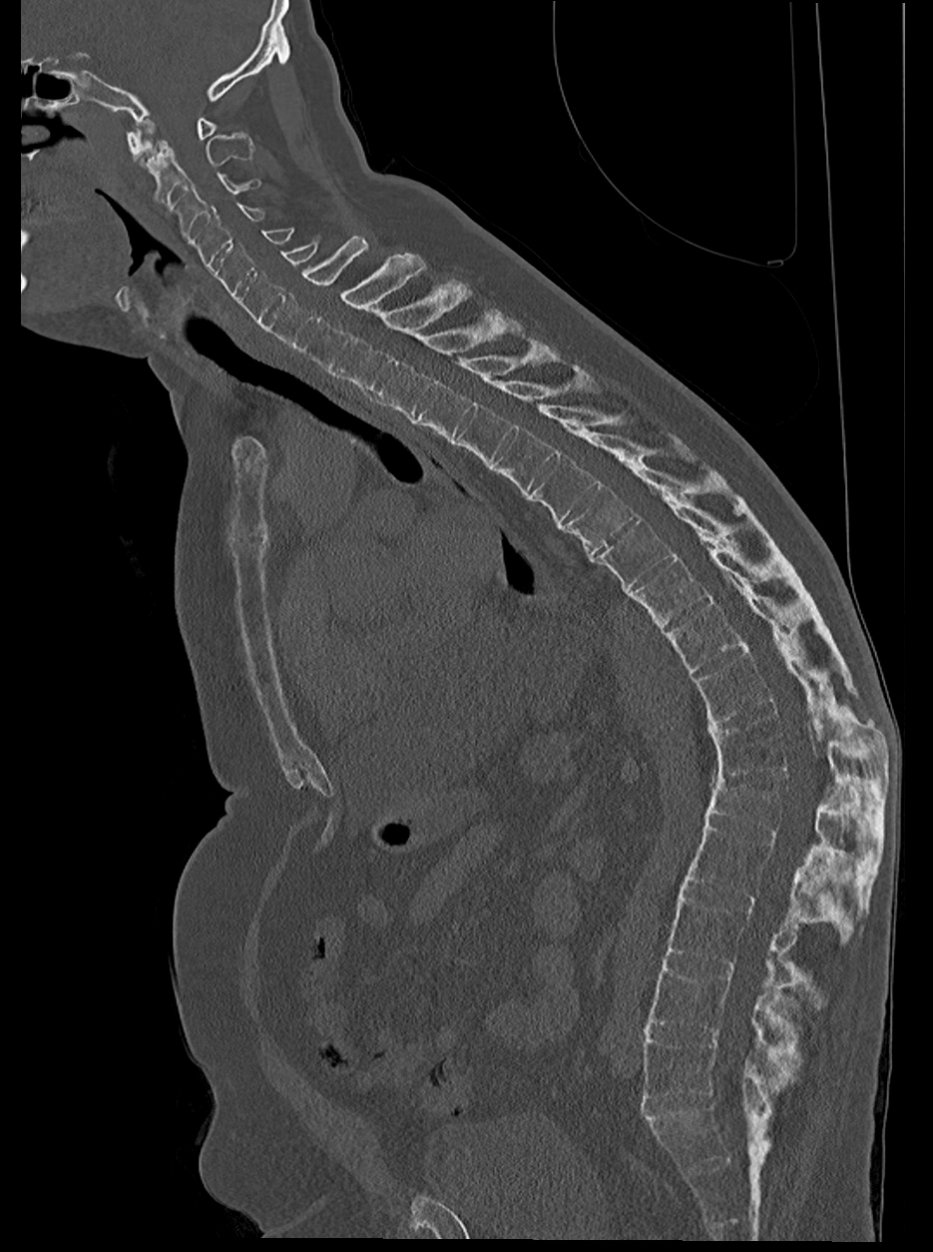
The CT scan with sagittal reconstruction shows the ossification and the global kyphosis of the whole spine and the old fracture at the level L1 healed in a kyphotic position.

The whole spine conventional X-rays in two planes revealed an ossification of the spine, reduced bone mineral density, a global kyphotic deformity with a sagittal imbalance and the kyphotic healed fracture at the level L1 (Figure 1 (Fig. 1)). The CT scan showed the typical complete ossification of the spine and the described kyphotic deformity (Figure 2 (Fig. 2)). The DEXA measurement showed a pronounced osteoporosis.

We planned to perform a pedicle subtraction osteotomy at the level of L3 to achieve a correction of about 30 degrees. 

The patient was prepared for surgery under consideration of the special features related to the underlying disease. After fiber optic intubation and treatment with single-shot antibiotic, the patient was careful positioned on the operating table. Therefore, the table was adapted to the patient’s body shape and the patient was positioned with several helpers under protection of the whole spine. 

**Figure 3:**
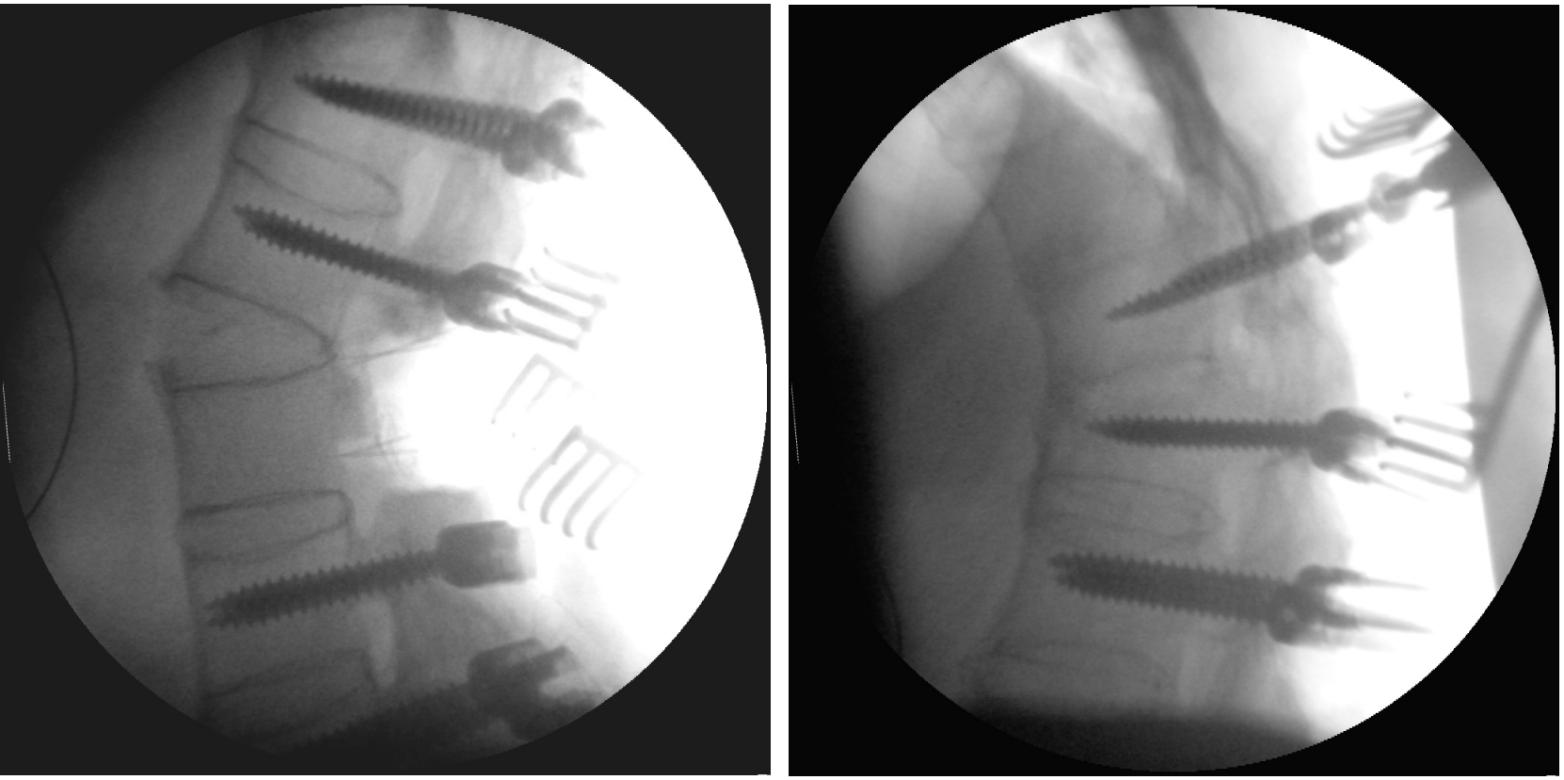
The intraoperatively taken fluoroscopy shows a fracture of the ankylosed disc L2/3 with a wedge-shaped gape without any translation or rotation (left). The fluoroscopic control performed a short time before to assess the right screw position shows the later fractured level without any particularity (right).

**Figure 4:**
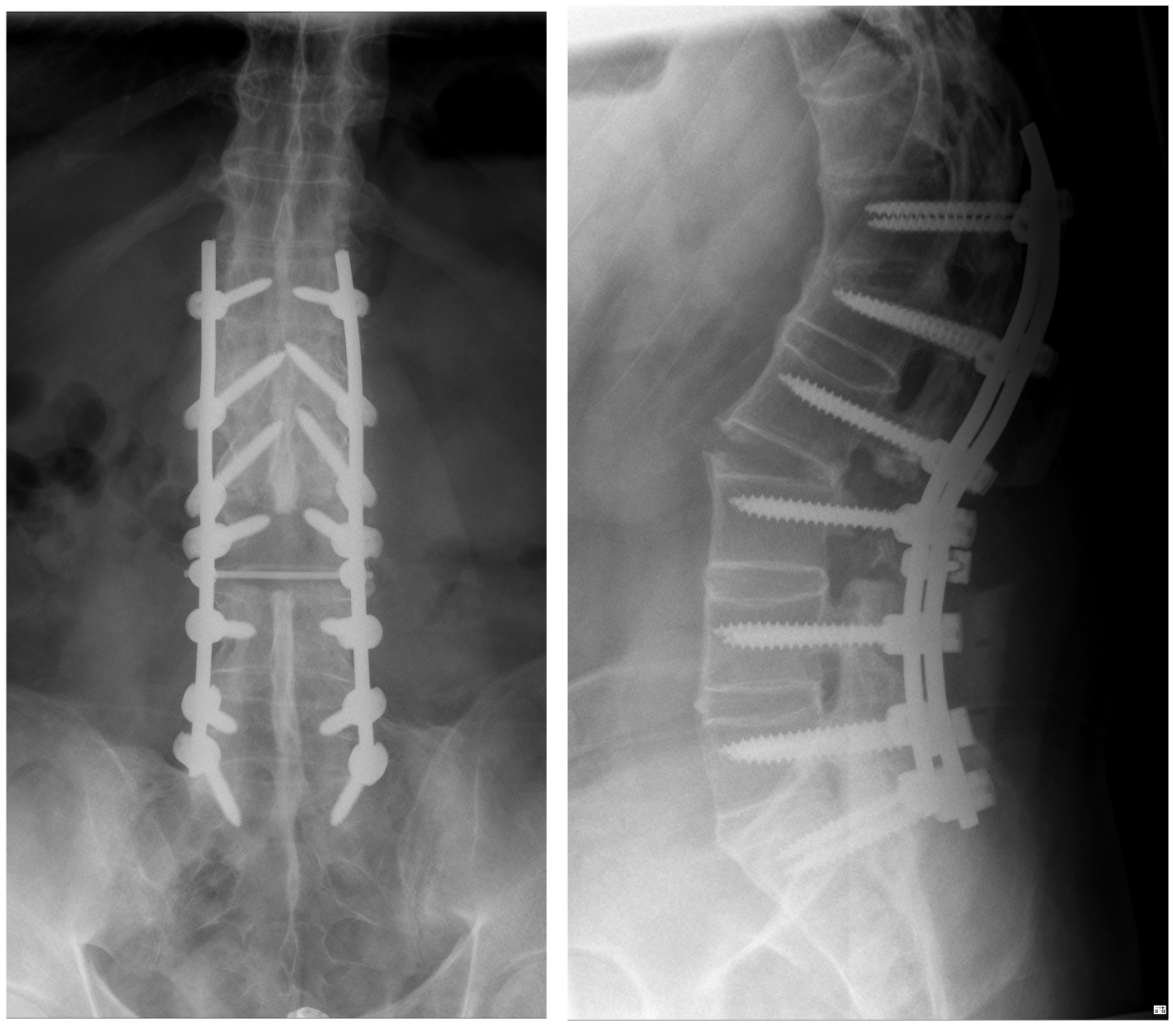
The conventional X-rays of the lumbar spine after the first operation in two planes (ap view left, lateral view right) show the improved position of the spine, however, compared to the intraoperative fluoroscopy (Figure 3), a partial loss of correction.

The first part of surgery was performed without any particularity. Screws were inserted and we started with the posterior part of the osteotomy. During this procedure, a change in the position of the spine (increase of lordosis) was observed. The immediately performed fluoroscopy showed a spontaneous fracture at the level of the ankylosed disc L2/3 (Figure 3 (Fig. 3)). It was a wedge-shaped fracture, fortunately without any rotation or translation. The resulting condition could be described as a Smith-Peterson osteotomy. Therefore, we decided not to carry out the initially planned osteotomy at level L3 but rather use the gap from the spontaneous fracture for correction. Thus, additional screws were inserted at the level of L3 and posterior instrumentation was completed. Furthermore, bone was replaced posteriorly to support fusion and surgery could be completed (Figure 4 (Fig. 4)). Postoperatively, we monitored the hemoglobin value and did repeated ultrasound examinations of the abdomen to exclude an impairment of the vessels in front of the lumbar spine.

**Figure 5:**
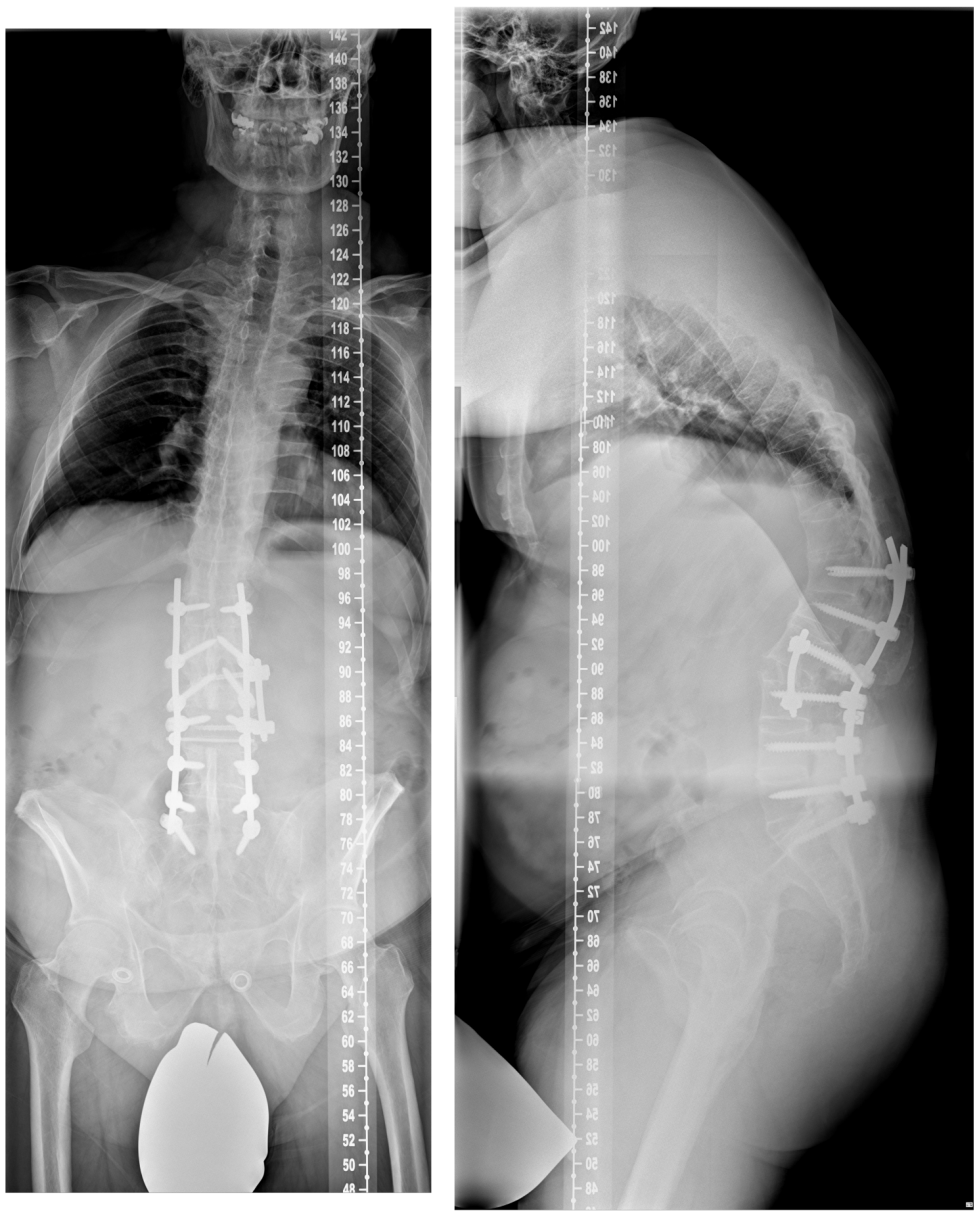
The whole spine conventional X-rays after the second surgery reveal an improved sagittal profile, however, the initially planned and desired amount of correction has not been reached completely.

In the follow-up, we discussed how to proceed and decided to perform an additional anterior surgery. The goal was to support the anterior column of the spine and to add stability to ensure healing in a good position. Furthermore, loss of correction was observed at the postoperative standing conventional lateral X-rays compared to the intraoperatively taken fluoroscopic images. This second surgery could be performed without any problem (Figure 5 (Fig. 5)). Wound healing was uneventful, and the patient was discharged in a good condition.

## Discussion

Ankylosing spondylitis leads to distinct changes of the biomechanical properties of the spinal column. Ossification with subsequent loss of mobility and progressive kyphosis with subsequent spinal imbalance as well as osteoporosis result in a significantly increased fracture risk particular to minor trauma [1], [2], [3]. The substantial changes of the spine with a resulting restriction of the normal visual axis may require corrective osteotomies [8], [9]. Different surgical techniques available today are well established. Both, the correction of the sagittal balance and particularly the restoration of the visual axis provide a sustainable improvement in the quality of life for this patient group [8], [9]. 

As mentioned above, these patients show a significantly higher risk for spinal fractures regarding to minor trauma [3], [4], [5], [6], [7]. Surgical procedures and the related perioperative measures pose such a risk [11]. This is related to the increased susceptibility of the spine due to the reduced bone quality and the changed lever arms. In addition, the loss of muscle tone under relaxation during surgery increases the risk.

It is well known that brusque maneuvers should be strictly avoided in this patient group. However, also manipulations with low forces during surgical procedures or positioning maneuvers seem to be suitable to cause a fracture. 

If these fractures are accompanied with lengthening of the anterior part of the spine, the possible impairment of the great vessels in front of the spine should be considered [12]. Regarding fractures in ankylosing spondylitis (acute or pathological fractures), there is an ongoing discussion, if the resulting instability should be used for corrective maneuvers during surgical stabilization procedures [13].

The presented case shows that despite cautious and careful handling the above-described fracture occurred. Another reason could be the required special position of the patient on the operating table. In case of closing wedge osteotomy techniques, the sagittal profile will be restored by shorting the spine via closing the wedge posteriorly. This maneuver leads to more lordosis which is the desired effect of this procedure. To realize this, the abdomen must be positioned on the operation table without support in the area where the correction will take place to allow for the planned correction. This could be one of the reasons that the spinal column, weakened by the described biomechanical changes, is at risk of fracture if surgical manipulations are performed in this area. 

In the presented case, no translation or rotation in the fracture was to observe. Thus, we were able to use the anterior opening of the spinal column for correction, even if different from planned. We adapted the surgical strategy intraoperatively to the new situation. However, we observed a partial loss of correction on the conventional X-rays postoperatively compared to the fluoroscopy taken intraoperatively. At this point it could be discussed whether a three or four-rod construct would be better to avoid loss of correction [8]. Finally, we performed an additional anterior surgery to secure both stability and fusion. The second approach might be debated; however, our goal was to obtain as much safety as possible and to avoid further loss of correction.

## Conclusion

The presented case emphasizes strongly that patients with ossification of the spinal column due to ankylosing spondylitis must be treated with the utmost caution. Surgical experiences in this field and familiarity with the underlying disease are essential to obtain good results and to manage unexpected complications. 

## Notes

### Competing interests

The authors declare that they have no competing interests.
